# Expression kinetics of natural resistance associated macrophage protein (NRAMP) genes in *Salmonella* Typhimurium-infected chicken

**DOI:** 10.1186/s12917-018-1510-4

**Published:** 2018-06-08

**Authors:** Mashooq Ahmad Dar, Raashid Ahmed, Uneeb Urwat, Syed Mudasir Ahmad, Pervaiz Ahmad Dar, Zahid Amin Kushoo, Tanveer Ali Dar, Peerzada Tajamul Mumtaz, Shakil Ahmad Bhat, Umar Amin, Nadeem Shabir, Hina Fayaz Bhat, Riaz Ahmad Shah, Nazir Ahmad Ganai, Mohammad Heidari

**Affiliations:** 1 Division of Biotechnology, Faculty of Veterinary Sciences and Animal Husbandry, Shuhama, Sher-e- Kashmir University of Agricultural Sciences and Technology – Kashmir, Srinagar, India; 2 Depatment of Biochemistry, University of Kashmir, Srinagar, India; 3 Division of Veterinary Microbiology, Faculty of Veterinary Sciences and Animal Husbandry, Shuhama, SKUAST-K, Srinagar, India; 4 Division of Veterinary Pathology, Faculty of Veterinary Sciences and Animal Husbandry, Shuhama, SKUAST-K, Srinagar, India; 5 Division of Animal Breeding and Genetics, Faculty of Veterinary Sciences and Animal Husbandry, Shuhama, SKUAST-K, Srinagar, India; 6 USDA, Agricultural Research Service, Avian Disease and Oncology Laboratory, 4279 E. Mount Hope Rd., East Lansing, MI 48823 USA

**Keywords:** *Salmonella* Typhimurium, Poultry, Histopathology, Biochemistry, Real time expression, NRAMP

## Abstract

**Background:**

*Salmonella enterica serovar* Typhimurium (*Salmonella* Typhimurium) is a zoonotic pathogen responsible for severe intestinal pathology in young chickens. Natural resistance-associated macrophage protein (NRAMP) family has been shown to be associated with resistance to intracellular pathogens, including *Salmonella* Typhimurium. The role of NRAMP proteins in macrophage defence against microbial infection has been ascribed to changes in the metal-ion concentrations inside the bacteria-containing phagosomes. The present study was conducted to investigate tissue-specific (liver, spleen and caecum) expression kinetics of NRAMP gene family (NRAMP1 and NRAMP2) in broilers from day 0 to day 15 after *Salmonella* Typhimurium challenge concomitant to clinical, blood biochemical and immunological parameters survey.

**Results:**

Clinical symptoms appeared 4 days post-infection (dpi) in infected birds. Symptoms like progressive weakness, anorexia, diarrhoea and lowering of the head were seen in infected birds one-week post-infection. On postmortem examination, liver showed congestion, haemorrhage and necrotic foci on the surface, while as the spleen, lungs and intestines revealed congestion and haemorrhages. Histopathological alterations were principally found in liver comprising of necrosis, reticular endothelial hyperplasia along with mononuclear cell and heterophilic infiltration. Red Blood Cell (RBC) count, Haemoglobin (Hb) and Packed Cell Volume (PCV) decreased significantly (*P* < 0.05) in blood while heterophil counts increased up to 7 days post-infection. Serum glucose, aspartate transaminase (AST) and alanine transaminase (ALT) enzymes concentrations increased significantly throughout the study. A gradual increase of specific humoral IgG response confirmed *Salmonella* infection. Meanwhile, expression of NRAMP1 and NRAMP2 genes was differentially regulated after infection in tissues such as liver, spleen and caecum known to be the target of *Salmonella* Typhimurium replication in the chicken.

**Conclusion:**

Thus the specific roles of NRAMP1 and NRAMP2 genes in Salmonella Typhimurium induced disease may be supposed from their differential expression according to tissues and timing after per os infection. However, these roles remain to be analyzed related to the severity of the disease which can be estimated by blood biochemistry and immunological parameters.

## Background

*Salmonella* is an intracellular pathogenic, gram negative, facultative anaerobe, non-spore forming, and usually motile bacilli that leads to salmonellosis in the host. Among the different diseases occurring in poultry, those caused by the genus *Salmonella* are the most common, causing serious economic losses to the poultry industry in terms of mortality, reduced growth and loss in egg production [1]. Salmonella infection can reach eggs either through vertical or horizontal transmission thus making it an important zoonotic disease [2]. Salmonellosis is a big socioeconomic threat worldwide that causes considerable mortality and morbidity in both humans and animals [3]. Owing to growing public health concern, prevention of foodborne transmission of *Salmonella* spp. remains one of the major foci for the poultry sector [4]. The gastrointestinal (GI) tract of newly hatched chickens is usually sterile and presents an empty ecological niche that provides easy access for the pathogen to colonize with limited restriction making them highly susceptible to enteric bacterial infections, such as *Salmonella* [5]. After ingestion, the bacteria can survive the acidic pH of the stomach and is taken via microfold (M)-cells and reach liver and spleen through the hepatic portal system where the bacteria multiply in macrophages of reticuloendothelial system [6]. Many genes such as Major Histocompatibility Complex (MHC), Caspase1, NRAMP Family, inducible nitric oxide synthase (iNOS), those encoding complement proteins and Toll-like Receptor 4 (TLR4) have been found to be associated with resistance against *Salmonella* infection in poultry [7].

The Solute Carrier Family (SLC11A), formerly known as natural resistance associated macrophage protein family functions as metal ion transporters in diverse organisms from bacteria to human [8]. Iron is essential for the survival of bacteria inside the macrophages and any impairment in iron homeostasis may promote inappropriate immune responses thereby affecting the host resistance to infectious diseases [9]. Iron transporter natural resistance-associated macrophage protein 1 (NRAMP1/SLC11A1), the member of the solute carrier (SLC11A) family of ion transporters [10] has certain genotypes that provide resistance to wide range of intracellular pathogens including *Salmonella* Typhimurium [11]. NRAMP1 is an antiporter, expressed on phagosomes and primary phagolysosomes [12] that transports iron into the phagosome [13], where it catalyzes the Haber-Weiss reaction [14]. This results in an increase in the production of highly microbicidal hydroxyl radicals that inhibit bacterial growth. SLC11A2 formerly known as NRAMP2/ DMT1 is ubiquitously expressed in all tissues including the brush border of intestinal epithelial cells and is involved in transport of divalent cations into the cytosol of cells as well as intestinal absorption of iron, while as NRAMP1 has more restricted expression but is especially associated with myeloid lineage cells and the liver, thymus and spleen [15–18]. Polymorphisms in the NRAMP1 gene have been found to be significantly associated with caecal *Salmonella* colonization, and expression of NRAMP1 gene can accelerate inflammatory responses in caecum [19]. NRAMP1 plays an important role in modulating the growth of bacteria that reach the reticuloendothelial system during early infection [20]. Association of NRAMP1 Ser^379^ polymorphism with *Salmonella* colonization of the spleen has been found in young chickens and also linkage analysis studies have shown that NRAMP1 is associated with resistance in *Salmonella* Typhimurium-infected chicken and mice [21–23]. NRAMP2 is expressed in almost all tissues in *Salmonella*-infected chicken [24]. However, detailed studies of expression kinetics of NRAMP genes in liver and spleen (that form the hepatoportal system) and caecum in chicken at different days post *Salmonella* Typhimurium infection has not been carried out. Thus, the present study was conducted to investigate the mRNA expression kinetics of NRAMP1 and NRAMP2 in liver, spleen and caecum of birds challenged with *Salmonella* Typhimurium in relation with blood physiological and immunological parameters along with the disease severity.

## Methods

### Experimental birds

Cobb breeding flock procured from the Hariparbat hatchery Srinagar, Department of Animal Husbandry government of Jammu and Kashmir, India, was maintained under strict hygienic conditions at the experimental housing of Faculty of Veterinary Science and Animal Husbandry, Sher-e-Kashmir University of Agricultural Sciences and Technology, Kashmir, India and the work was agreed upon by the Institutional Animal Ethics Committee on ethical standards in animal experimentation (No: AU/FVS/PS-57/4461) (IAEC/15/01).

### *Salmonella* Typhimurium strain

For the development of infection in experimental birds, *Salmonella* Typhimurium was isolated in the field from fecal and liver samples of salmonellosis-suspected chicks at the Division of Veterinary Pathology, F.V.Sc and AH, Shuhama, Srinagar Jammu and Kashmir-India. Samples were inoculated in tetrathionate broth (TTB) and incubated at 37 °C for 48 h. The cultures were identified by a method as recommended [25]. The *Salmonella* Typhimurium was confirmed by PCR as described previously [26] and biochemical (IMViC) tests. For further confirmation serotyping was done at National Salmonella and Escherichia Centre (NSEC), Himachal Pradesh, India.

### Experimental design

A total of 170 one day-old chickens were divided into two groups (control and infected) and were checked to be free of *Salmonella* infection before the challenge. After three days of acclimatization, the chicks in the infected group were challenged orally with 2 × 10^8^ CFU of *Salmonella* Typhimurium. Chicks in the control group were given 1 ml of nutrient broth.

### Confirmation of infection

Faecal swabs were taken 12 h after infection and cultured in TTB broth and incubated for 18 h at 37 °C. The growth of TTB broth was streaked on BGA and MacConkey agar. These plates were incubated for 24–48 h at 37 °C. The plates were examined for typical colonies of *Salmonella* i.e. pale colonies on MacConkey agar, pink colonies on Brilliant Green Agar (BGA).

### Sampling

The birds were euthanized with CO_2_ and subsequently sharp cut was made in the neck to drain blood from vessels. Blood samples were collected for blood cell enumeration and biochemical analysis, and organs (liver, spleen and caecum) were collected for histopathological and gene expression studies from 6 birds randomly selected in each group at 0, 1, 3, 5, 7, 9, 11, 13 and 15 days post infection. For real time gene expression, tissue samples were collected in Trizol (Invitrogen, USA) and stored at − 80 °C for RNA extraction. The blood samples were analyzed for haemoglobin (Hb), packed cell volume (PCV), red blood cell (RBC) count, white blood cell (WBC) count, heterophils and lymphocyte count using Sysmex (KX-21) automated haematology analyzer. Serum samples were analyzed for alanine transaminase (ALT), aspartate transaminase (AST), total protein, serum albumin and glucose using commercially available kits.

### Histopathology

For histopathological studies, representative liver tissue samples from infected and control birds were collected and fixed in 10% neutral buffered formalin. These were processed for paraffin embedding using alcohol as dehydrating agent and benzene as the clearing agent. The sections were cut at 4–5 μm thickness and stained by the routine haematoxylin and eosin method [27].

### Enzyme linked immunosorbent assay (ELISA)

Indirect ELISA was performed to detect the anti-Salmonella IgG antibodies in serum as per the method described [28]. Sonicated *Salmonella* Typhimurium (20 ng/100 μl) was used as antigen. One hundred μl of goat anti-chicken antibody (Sigma 1:15000) diluted in PBS-T (Phosphate buffered saline -tween) was added to each well and plate was incubated at 37 °C for 2 h. Plates were washed with PBS Tween-20 (0.05%) and incubated with 100 μl O-phenylene diamine dihydrochloride in the dark at room temperature for 15 min. The reaction was stopped by adding 2 N H_2_SO_4_ @ 50 μl per well. Absorbance was determined using ELISA reader (PerkinElmer) at 492 nm. Known positive (*n* = 5) and known negative (*n* = 22) controls were used in this study. The specificity was statistically calculated as.

Specificity = T_N_/T_N_ + F_P_.

Where T_N_ denotes total negatives and F_P_ denotes false positives.

### Gene expression analysis

#### RNA extraction

Total RNA was extracted from the tissue samples by Trizol method (Invitrogen, USA) as per manufacturer’s protocol. The quantity and quality of isolated RNA were checked at 260 and 280 nm with UV–Visible spectrophotometer. Prior to cDNA synthesis, RNA samples were run on 1% agarose gel. DNase treatment using DNase1 kit (Sigma, USA) was given to rule out any genomic DNA contamination.

#### cDNA synthesis

cDNA synthesis was done with an equal concentration of RNA (1.5 μg) in all the samples using Thermo Scientific RevertAid First Strand cDNA Synthesis Kit™ (Lithuania) using oligo dT primers following manufacturer protocol. In conventional PCR, all primers produce only one amplification band visualized by agarose gel electrophoresis, indicating the specificity of the amplification. The primers used for NRAMP1 and β-actin gene amplification were already reported [24]. Primers for NRAMP2 were designed using PRIMER 3 Plus. (Table 1).

**Table 1:** List of primers used for real time PCR

Target	Primer sequence (5′ → 3′)		Size of Amplicon	T_m °C_	Reference
*β Actin*	Forward primer	TGGCATTGCTGACAGGAT	160 bp	63.1	[24]
	Reverse primer	CTGCTTGCTGATCCACAT		60.0	
*NRAMP1*	Forward primer	CCCCCACATCACCCCGTCC	180 bp	74.2	[24]
	Reverse primer	GGCCCCACACTGCAGGTCTGAC		74.4	
*NRAMP2*	Forward primer	GTACTCAGGGCAGTTCGTCA	162 bp	63.0	This study
	Reverse primer	GCACGTTGAGGAAGTCGTTC		64.8	

#### Quantitative PCR

For real time PCR, β-actin was used as internal control and SYBR green as fluorescent dye to quantify the mRNA level of chicken NRAMP1 and NRAMP2 genes by RT-PCR analysis. To confirm reproducibility, all determinations were performed at least two times. The reaction mixture contained 10 μL 2X Q-PCR SYBR Green Mix (Roche), 0.3 μL 10 μM of each primer, 8.9 μL ultrapure RNase-free water, and 0.5 μL cDNA to a final volume of 20 μL. Running program was performed in a Light cycler 480 II (Roche). All Aliquots were then amplified by 40 cycles of denaturation at 95 °C for 5 min, annealing at 60 °C for 15 s and extension at 72 °C for 15 s. Melting curve analysis was performed to confirm the specificity of the product. NRAMP1 and NRAMP2 mRNA relative expression was indicated by 2^-∆∆C^_T_, where ∆∆C_T_ corresponded to the difference between the C_T_ measured for the mRNA level of each tissue and the C_T_ measured for the mRNA level of the reference tissue, ∆C_T_ = C_T_ (target gene) - C_T_ (β-actin).

### Statistical analysis

The data obtained in the current study was analyzed using a computer-aided statistical software package SPSS 20.0. The differences between means were analyzed by unpaired Student′s t-test and by one way ANOVA followed by Fisher′s LSD test and significance was determined at *P* < 0.05.

## Results

### Clinical signs

After inoculating 2 × 10^8^ CFU of *Salmonella* Typhimurium, clinical symptoms appeared within 4 dpi and included dullness, depression, inappetence and reluctance to move with both eyes closed. After one week of infection, birds showed progressive weakness, anorexia, increased thirst, diarrhoea, dropping of wings, ruffled feathers and lowering of the head.

### Gross pathology

The birds in the control group did not exhibit any gross lesion in any of the organs examined throughout the experimental trial. However, in infected birds, liver revealed severe congestion, haemorrhages and enlargement. Congestion of liver was observed up to 15 dpi. Formation of pinpoint foci on the surface of the liver was observed at 9 dpi. Spleen and lungs of infected birds revealed congestion and haemorrhages from 1 to 15 dpi. Mild catarrhal enteritis, congestion and severe haemorrhages throughout the intestinal tract were noticed. Mostly, severe haemorrhages were observed in the second week post-infection.

### Histopathology

**Fig. 1:**
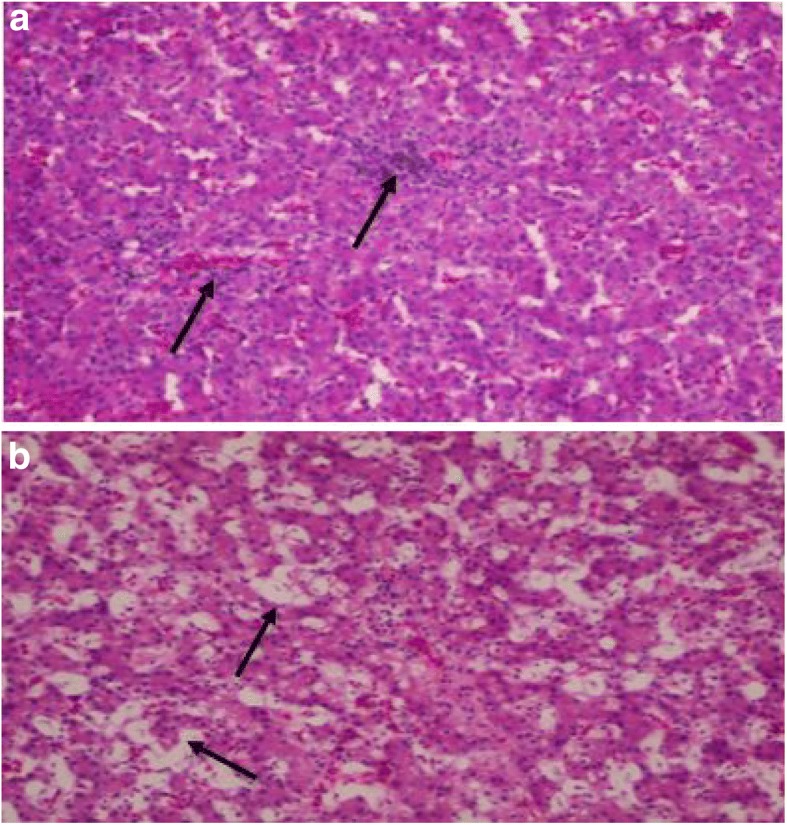
**a** Histopathological examination of liver showing leukocytic infiltration of the infected birds at 13 dpi. H&E × 10. **b** Histopathological examination showing hepatic parenchyma degeneration in infected birds at 13 dpi. H&E × 10

Liver of birds infected with *Salmonella* Typhimurium revealed congestion of blood vessels at all time points. Chicks mostly showed degeneration of hepatic parenchyma along with distortion of hepatic cords and leukocytic infiltration comprising of lymphocytes and heterophils at 13 dpi (Fig. 1a, b).

### Haematology

The haematological results are presented in Table-2. The mean values of Hb, RBC and PCV were significantly lower (*P < 0.05*) in the infected group as compared to control group starting day 1 post-infection. There was a significant decrease in the lymphocyte count in the infected birds as compared to their respective controls (*P < 0.05*). After an initial decrease, recovery in the number of circulating lymphocytes in infected birds was observed. Further there was a significant increase in the heterophil count in the infected birds compared to their respective controls up to day 5 post- infection, followed by a gradual increase thereafter. (Table 2).

**Table 2:** Effect of *Salmonella* Typhimurium infection on haematological parameters at different days post infection (dpi)

Parameter	Group (Control (*N* = 6) (Infected (*N* = 6)	0 (dpi)	1 (dpi)	3 (dpi)	5 (dpi)	7 (dpi)	9 (dpi)	11 (dpi)	13 (dpi)	15 (dpi)
RBC (10^6^/μl)	Control	1.745^a^ ± 0.024	1.860^a^ ± 0.021	1.941^a^ ± 0.025	2.031^a^ ± 0.024	2.045^a^ ± 0.038	2.108^a^ ± 0.023	2.155^a^ ± 0.032	2.228^a^ ± 0.032	2.265^a^ ± 0.021
	Infected	1.723^a^ ± 0.008	1.700^b^ ± 0.020	1.671^b^ ± 0.014	1.655^b^ ± 0.024	1.623^b^ ± 0.100	1.585^b^ ± 0.009	1.615^b^ ± 0.023	1.633^b^ ± 0.013	1.658^b^ ± 0.027
Haemoglobin (g/dl)	Control	10.018^a^ ± 0.226	10.621^a^ ± 0.118	11.04^a^ ± 0.13	11.790^a^ ± 0.147	12.011^a^ ± 0.126	12.343^a^ ± 0.059	12.516^a^ ± 0.060	12.553^a^ ± 0.104	12.800^a^ ± 0.064
	Infected	10.001^a^ ± 0.211	9.368^b^ ± 0.078	9.041^b^ ± 0.074	8.880^b^ ± 0.105	8.806^b^ ± 0.074	8.675^b^ ± 0.070	8.791^b^ ± 0.023	8.830^b^ ± 0.038	8.963^b^ ± 0.063
PCV (%)	Control	26.656^a^ ± 0.055	26.855^a^ ± 0.076	27.29^a^ ± 0.070	27.590^a^ ± 0.123	27.805^a^ ± 0.143	28.080^a^ ± 0.061	28.258^a^ ± 0.057	28.628^a^ ± 0.109	28.990^a^ ± 0.170
	Infected	26.605^a^ ± 0.042	26.263^b^ ± 0.053	25.91^b^ ± 0.113	25.743^b^ ± 0.079	25.470^b^ ± 0.057	25.141^b^ ± 0.080	25.365^b^ ± 0.059	25.408^b^ ± 0.048	25.446^b^ ± 0.063
WBC (10^3^/μl)	Control	20.125^a^ ± 0.390	20.828^a^ ± 0.385	21.48^a^ ± 0.325	21.675^a^ ± 0.276	21.730^a^ ± 0.599	22.038^a^ ± 0.720	22.396^a^ ± 0.324	23.010^a^ ± 0.474	23.160^a^ ± 0.426
	Infected	20.303^a^ ± 0.248	27.085^b^ ± 0.300	32.76^b^ ± 0.399	38.368^b^ ± 0.431	36.160^b^ ± 0.318	34.060^b^ ± 0.320	32.556^b^ ± 0.486	27.980^b^ ± 0.444	25.046^b^ ± 0.416
Heterophils (10^3^/μl)	Control	10.545^a^ ± 0.079	10.46^a^ ± 0.077	10.35^a^ ± 0.065	10.293^a^ ± 0.059	10.183^a^ ± .037	10.030^a^ ± 0.047	9.676^a^ ± 0.100	9.393^a^ ± 0.064	9.180^a^ ± 0.036
	Infected	10.531^a^ ± 0.079	15.055^b^ ± 0.100	21.32^b^ ± 0.038	26.146^b^ ± 0.018	23.205^b^ ± 0.017	20.465^b^ ± 0.032	18.140^b^ ± 0.033	17.185^b^ ± 0.027	15.963^b^ ± 0.161
Lymphocytes (10^3^/μl)	Control	6.888^a^ ± 0.010	7.170^a^ ± 0.020	7.330^a^ ± 0.012	7.518^a^ ± 0.009	7.796^a^ ± 0.014	8.170^a^ ± 0.017	8.340^a^ ± 0.020	8.513^a^ ± 0.014	8.930^a^ ± 0.050
	Infected	6.873^a^ ± 0.026	6.583^b^ ± 0.208	5.755^b^ ± 0.318	4.970^b^ ± 0.110	8.453^b^ ± 0.178	10.773^b^ ± 0.279	12.833^b^ ± 0.335	15.188^b^ ± 0.362	17.115^b^ ± 0.471

Values are presented as mean ± SE

Values having different superscript (a and b) differ significantly (*P* < 0.05)

Number of birds from each group that were evaluated at each time point (dpi) was 6, each sample was run in duplicates. Statistical analysis was done using unpaired Student′s t-test and by one way ANOVA followed by Fisher′s LSD test

### Serum biochemical profile

The mean values of glucose levels were significantly elevated in the infected birds when compared to their respective controls starting day 1 post-infection. Serum protein contents were lower in the infected group when compared with corresponding values in the control group after day 0 post-infection, as shown in Table 3. Similarly, mean values of serum albumin were significantly lower (*P < 0.05*) in the infected group at almost all intervals except at 0 dpi where no significant changes were seen. The AST and ALT in the infected group showed a sharp and significant increase (*P < 0.05*) when compared to their respective controls from day 1 post infection till the end of the experiment (Table 3).

**Table 3:** Effect of *Salmonella* Typhimurium infection on serum biochemical parameters at different days post infection (dpi)

Parameter	Group (Control (*N* = 6) (Infected (*N* = 6)	0 (dpi)	1 (dpi)	3 (dpi)	5 (dpi)	7 (dpi)	9 (dpi)	11 (dpi)	13 (dpi)	15 (dpi)
ALT (IU/L)	Control	8.98^a^ ± 0.097	9.27^a^ ± 0.123	9.40^a^ ± 0.135	9.63^a^ ± 0.138	9.76^a^ ± 0.097	10.05^a^ ± 0.085	10.19^a^ ± 0.109	10.43^a^ ± 0.124	10.87^a^ ± 0.175
	Infected	9.08^a^ ± 0.138	25.62^b^ ± 0.221	45.60^b^ ± 0.380	63.90^b^ ± 0.219	67.88^b^ ± 0.237	73.96^b^ ± 0.317	78.34^b^ ± 0.218	82.41^b^ ± 0.276	86.28^b^ ± 0.364
AST (IU/L)	Control	39.20^a^ ± 0.127	39.99^a^ ± 0.282	41.08^a^ ± 0.388	42.94^a^ ± 0.178	42.94^a^ ± 0.452	43.45^a^ ± 0.405	44.34^a^ ± 0.261	45.45^a^ ± 0.332	46.06^a^ ± 0.251
	Infected	39.77^a^ ± 0.177	44.48^b^ ± 0.638	49.73^b^ ± 0.779	54.46^b^ ± 0.910	67.27^b^ ± 1.136	77.81^b^ ± 0.438	90.26^b^ ± 0.535	108.44^b^ ± 0.763	120.81^b^ ± 0.862
Albumin (g/dl)	Control	0.848^a^ ± 0.006	0.888^a^ ± 0.004	0.901^a^ ± 0.003	0.927^a^ ± 0.003	0.964^a^ ± 0.002	0.985^a^ ± 0.003	1.011^a^ ± 0.006	1.063^a^ ± 0.008	1.111^a^ ± 0.013
	Infected	0.839^a^ ± 0.005	0.837^b^ ± 0.001	0.845^b^ ± 0.001	0.851^b^ ± 0.001	0.871^b^ ± 0.001	0.891^b^ ± 0.002	0.911^b^ ± 0.004	0.932^b^ ± 0.002	0.950^b^ ± 0.001
Protein (g/dl)	Control	1.698^a^ ± 0.004	1.756^a^ ± 0.006	1.876^a^ ± 0.008	1.990^a^ ± 0.012	2.051^a^ ± 0.013	2.150^a^ ± 0.005	2.215^a^ ± 0.008	2.365^a^ ± 0.007	2.543^a^ ± 0.014
	Infected	1.680^aa^ ± 0.003	1.726^b^ ± 0.004	1.745^b^ ± 0.008	1.798^b^ ± 0.009	1.816^b^ ± 0.009	1.861^b^ ± 0.003	1.925^b^ ± 0.004	1.981^b^ ± 0.007	2.021^b^ ± 0.017
Glucose (mg/dl)	Control	145.31^a^ ± 0.672	155.94^a^ ± 0.407	162.1^a^ ± 0.667	170.44^a^ ± 0.322	195.60^a^ ± 0.440	210.54^a^ ± 0.211	235.52^a^ ± 0.588	256.00^a^ ± 0.470	275.84^a^ ± 0.741
	Infected	148.36^a^ ± 0.297	170.89^b^ ± 0.672	178.7^b^ ± 0.759	197.51^b^ ± 0.634	211.34^b^ ± 0.978	232.04^b^ ± 0.293	247.13^b^ ± 0.694	269.92^b^ ± 0.297	290.03^b^ ± 0.752

Values are presented as mean ± SE

Values having different superscript (a and b) differ significantly (*P* < 0.05)

Number of birds from each group that were evaluated at each time point (dpi) was 6, each sample was run in duplicates. Statistical analysis was done using unpaired Student′s t-test and by one way ANOVA followed by Fisher′s LSD test

### Elisa

**Fig. 2:**
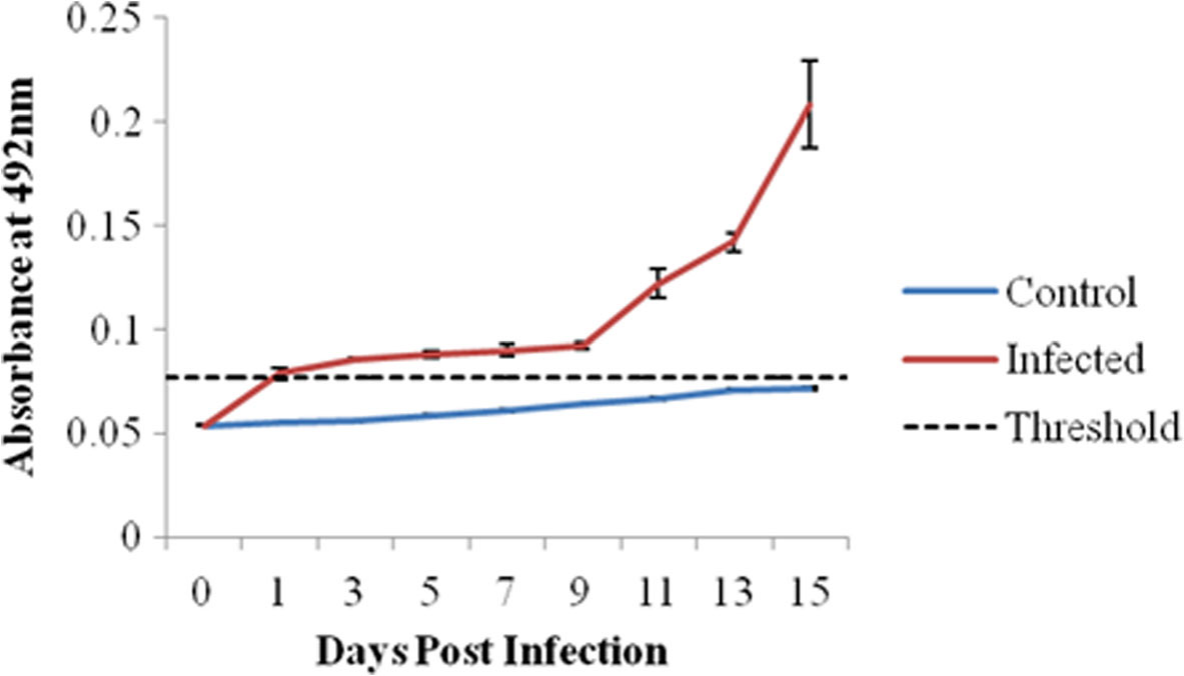
Level of Salmonella Typhimurium specific IgG response (mean OD ± SEM) detected in sera of Salmonella Typhimurium infected chicks and their respective uninfected controls at 0,1,3,5,7,9,11,13 and 15 days post infection (dpi). Number of birds from each group that were evaluated at each time point (dpi) was 6, each sample was run in duplicates. Statistical analysis was done using unpaired Student′s t-test and by one way ANOVA followed by Fisher′s LSD test

Specific serum (IgG) antibody response to *Salmonella* Typhimurium as the mean OD values of the chicks challenged orally with *Salmonella* Typhimurium increased from 0.0545 to 0.2088 up to 15 dpi as shown in Fig. 2. The overall antibody (IgG) response of the infected group was found to be statistically significant (*P* < 0.05). The mean OD values of the infected group were higher as compared to their respective controls.

### NRAMP gene family mRNA expression

**Fig. 3:**
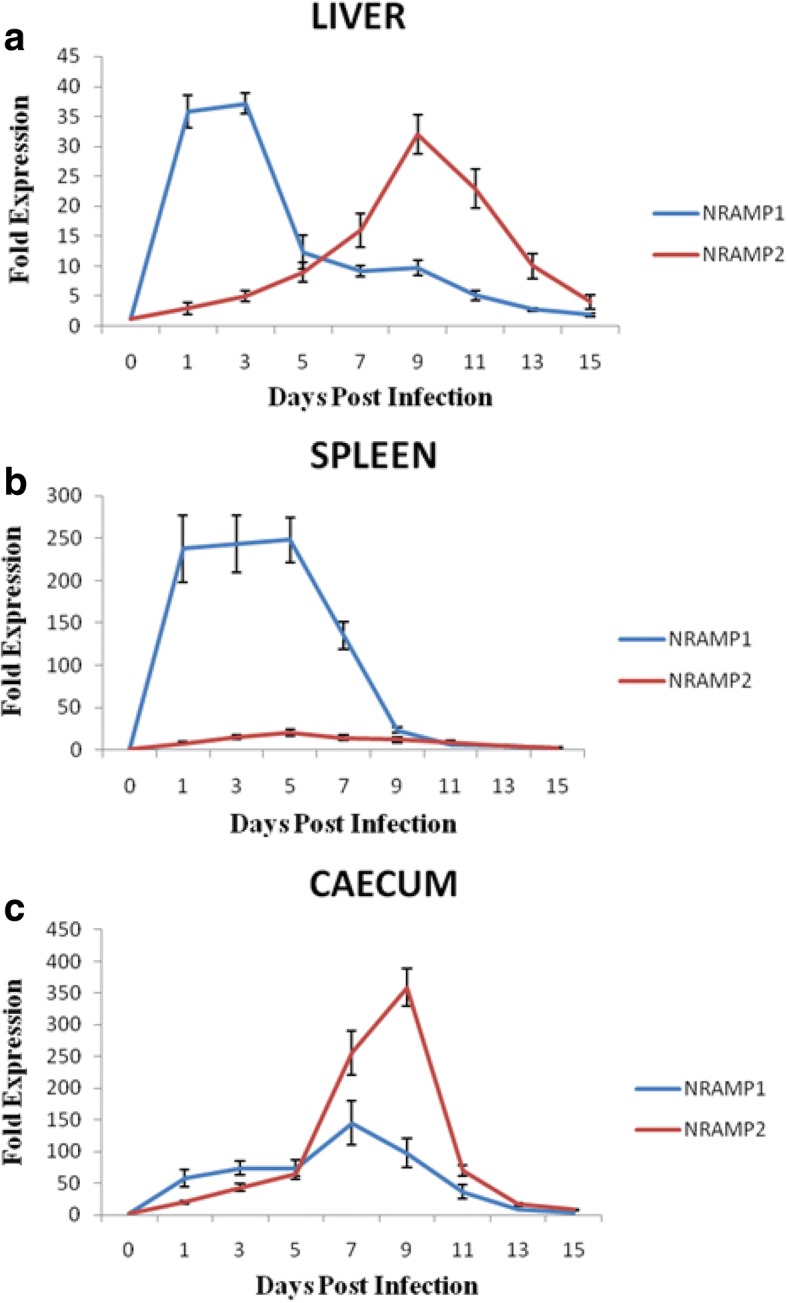
**a** mRNA expression of NRAMP1 and NRAMP2 at 0,1,3,5,7,9,11,13 and 15 days post infection (dpi) in liver of birds infected with *Salmonella* Typhimurium. The values are expressed as mean fold expression±standard error of mean. Number of birds from each group that were evaluated at each time point (dpi) was 6, each sample was run in duplicates. Statistical analysis was done using unpaired Student′s t-test and by one way ANOVA followed by Fisher′s LSD test. **b** mRNA expression of NRAMP1 and NRAMP2 at 0,1,3,5,7,9,11,13 and 15 days post infection (dpi) in spleen of birds infected with *Salmonella* Typhimurium. The values are expressed as mean fold expression±standard error of mean. Number of birds from each group that were evaluated at each time point (dpi) was 6, each sample was run in duplicates. Statistical analysis was done using unpaired Student′s t-test and by one way ANOVA followed by Fisher′s LSD test. **c** mRNA expression of NRAMP1 and NRAMP2 at 0,1,3,5,7,9,11,13 and 15 days post infection (dpi) in caecum of birds infected with *Salmonella* Typhimurium. The values are expressed as mean fold expression±standard error of mean. Number of birds from each group that were evaluated at each time point (dpi) was 6, each sample was run in duplicates. Statistical analysis was done using unpaired Student′s t-test and by one way ANOVA followed by Fisher′s LSD test

The mRNA expression levels of NRAMP1 and NRAMP2 increased significantly at all time intervals starting day 1post-infection. Following infection with *Salmonella* Typhimurium, expression of NRAMP1 mRNA was significantly up-regulated in all the three tissues (liver, spleen, caecum) compared to control group. Peak expression of NRAMP1 was found on day 3 in liver (37 fold, *P < 0.05*) (Fig. 3a), day 5 in spleen (247 fold, *P < 0.05*) (Fig. 3b) and on day 7 in caecum (144 fold, *P < 0.05*) (Fig. 3c). Similarly, expression of NRAMP2 mRNA was significantly up-regulated in infected birds from day 1 up to day 9 (32 fold, *P* < 0.05) in liver (Fig. 3a), up to day 5 (20 fold, *P* < 0.05) in the spleen (Fig. 3b) and up to day 9 (359 fold, *P* < 0.05) in caecum (Fig. 3c). After day 9 in liver and caecum and day 5 in the spleen, NRAMP2 mRNA started returning to basal levels in the infected birds.

## Discussion

Although expression pattern of disease resistance genes against *Salmonella* Typhimurium infection has been studied in mammals [8, 19, 22], information regarding tissue-specific expression of these genes is poorly available in poultry. The non-specific clinical observations that we observed in our study were similar to the earlier studies [29, 30] and clinical signs of ruffled feathers, dullness and diarrhoea were also found in Fowl typhoid outbreak [31]. It was also reported in *Salmonella* Typhimurium infection, chicken showed high mortalities, decreased body weight, lameness, inappetence, conjunctivitis, respiratory manifestation, and pasty diarrhoea [32]. In post-mortem examination of infected birds, we observed congestion and haemorrhages in spleen and lungs, whereas necrosis, congestion and haemorrhages followed by leukocytic infiltration primarily seen in liver, as also reported previously [33, 34]. Small intestines revealed mild catarrhal enteritis, congestion and severe haemorrhages mostly in the second week of infection, as that of earlier reports [35, 36].

Haematological profile in animals is an important indicator of the physiological or pathophysiological status of the body [37]. In the present study, we observed that the mean haematological values of Hb, RBC and PCV decreased markedly after *Salmonella* Typhimurium infection. Similar findings were also reported in Japanese quail infected with Salmonella enteric Serovar Gallinarum [38]. The mean values of Hb and PCV showed marked decrease from day 0 to day 9 post-infection in infected group. As with PCV, younger chicken have lower total erythrocyte count and mean corpuscular heamoglobin (MCH) than adult ones [39]. A decrease in Hb and PCV in broiler chicken infected with Fowl typhoid was also reported earlier [33]. Heterophils display high phagocytic and killing activity. Thus an increase of blood heterophil number is an indication of acute infection and tissue damages [39]. Our observations related to heterophil and lymphocyte counts coincide with earlier studies for increased blood heterophils and lymphocytes counts in Fowl typhoid infection [33].

Serum biochemistry profiling is a major tool to identify the occurrence of acute infection in birds and mammals to predict the severity of disease [40]. The increase in serum glucose of infected chickens is possibly due to increased expression of glucose transporters in intestinal tract plus more glucose consumption for bacterial replication within macrophages [41, 42]. A decrease in serum protein and albumin levels has also been reported in chicken infected with acute fowl typhoid [33, 43]. The significant decrease in protein and albumin could be due to severe liver and kidney damage of infected birds that resulted in the failure of synthesis of plasma proteins and protein loss or decreased appetite [44]. Elevation in AST and ALT in infected birds is possibly due to hepatocellular damage with alteration in cell membrane permeability and leakage of cytoplasmic ALT into the blood [45]. Elevation in AST levels has also been reported in fowl typhoid infection of chicken [33].

Strong antigen-specific cell and humoral immune responses have both been temporally linked to clearance of *Salmonella* Typhimurium infection in chicks [46]. Oral infection of chickens with *Salmonella* Typhimurium stimulates systemic immunity and a significant increase of specific humoral response after 10 days post *Salmonella* Typhimurium infection was found in the present study. These results are in conformity with other studies showing a steady increase in specific IgG and/or IgA starting one week after infection in chickens with *Salmonella* Enteritidis [47] and *Salmonella* Typhimurium [48].

In the present study, during the early stages of *Salmonella* Typhimurium infection, mRNA expression of NRAMP1 upregulated significantly in all the three tissues under study which dropped to the basal expression levels thereafter. NRAMP1 has a major effect on survival of chicken during the first phase of *Salmonella* Typhimurium infection [22]. Previous studies using mouse models have shown that NRAMP1 is involved in the control of exponential growth of bacteria in reticuloendothelial organs and has also been found to be involved in *Salmonella* clearance [20, 49]. Our findings were in accordance with the earlier studies that showed that there occurs a rapid increase in NRAMP1 expression in the lamina propia of caecum following infection with *Salmonella* Typhimurium and further suggesting a role of NRAMP1 in the early inflammatory response [50]. NRAMP genes often show tissue-specific expression [51]. In the present study, the NRAMP2 mRNA expression showed an initial upregulation in all the tissues of infected birds under study. In agreement with our results, an increased mRNA expression of both NRAMP2 and NRAMP1 following infection of macrophages with the intracellular bacterium *Mycobacterium avium* has been found [22]. Also, lipopolysaccharide (LPS) stimulation resulted in increased NRAMP2 mRNA expression in mice macrophages [52]. NRAMP2 is expressed in the intestinal tract where it plays an important role in iron uptake and in liver NRAMP2 is responsible for iron transport from endosomes into the intracellular space [53, 54]. Unlike earlier studies on Partridge chicken, where the expression of NRAMP genes was found predominantly in liver and spleen compared to caecum [24], we found mRNA expression of NRAMP1 and NRAMP2 in salmonella-infected birds was predominant in caecum which was followed by liver and spleen. The differences of expression levels in various tissues could be due to the higher colonization of *Salmonella* Typhimurium in lower GIT followed by dispersion to peripheral organs and response to defence signals in various tissues. NRAMP genes have an important role in iron homeostasis as discussed in the background of the manuscript, so evaluating the role of NRAMP family in the regulation of serum and cellular iron in a state of bacterial infection can open new avenues to investigate the role of iron metabolism in bacterial infection in the chicken model.

## Conclusion

In conclusion, the significant increase in mRNA expression of NRAMP genes in caecum, liver and spleen of the infected chicken could enhance our understanding the importance of NRAMP genes in salmonellosis. With further well designed studies and thorough validation, the present study may serve as a base in development of blood markers for genetic selection of *Salmonella* resistant chicken with respect to NRAMP polymorphisms and iron metabolism. Additionally, blood investigations for biochemical and haematological parameters may aid in monitoring the physiological and pathological changes in *Salmonella* infection in poultry.

## Abbreviations

ALT: Alanine transaminase
ANOVA: Analysis of variance
AST: Aspartate transaminase
cDNA: Complementary deoxyribonucleic acid
CFU: Colony forming unit
CO_2_: Carbon dioxide
C_T_: Threshold value
DMT: Divalent metal ion transporter
dpi: Days post infection
FP: False positives
GIT: Gastrointestinal tract
Hb: Haemoglobin
Ig: Immunoglobulin
LPS: Lipopolysaccharide
LSD: Least square difference
MCH: Mean corpuscular heamoglobin
mRNA: Messenger ribonucleic acid
NRAMP: Natural resistance-associated macrophage protein
NSEC: National Salmonella and Escherichia Centre
OD: Optical density
PBS-T: Phosphate buffered saline
PCV: Packed cell volume
RBC: Red blood cells
RNA: Ribonucleic acid
SLC: Solute carrier
SLC: Solute carrier
SPSS: Statistical package for social sciences
TN: Total negatives
WBC: White blood cell
